# Supplementary material for: Examining the Interrelationships Between Social Isolation and Loneliness and Their Correlates Among Older British Adults Before and During the COVID-19 Lockdown: Evidence From Four British Longitudinal Studies
Source: Innov Aging. 2023 Nov 7;8(1):igad126. doi: 10.1093/geroni/igad126 (PMC10798826; doi:10.1093/geroni/igad126)
Supplement: igad126_suppl_Supplementary_Files_S1-S4 [file igad126_suppl_supplementary_files_s1-s4.docx]

*Innovation in Aging* Online Supplementary Material: Rosie Mansfield, Giorgio Di Gessa, Kishan Patel, Eoin McElroy, Jaques Wels, Morag Henderson, Jane Maddock, Jean Stafford, Andrew Steptoe, Marcus Richards, & Praveetha Patalay. Examining the inter-relationships between social isolation and loneliness and their correlates among older British adults before and during the COVID-19 lockdown: Evidence from four British longitudinal studies.

**Supplementary file S1. Demographic, socio-economic and health characteristics of the analytic samples**

| **Variable** | **NSHD** | | **NCDS** | | **BCS** | | | **ELSA** | |
| --- | --- | --- | --- | --- | --- | --- | --- | --- | --- |
|  | **t-2** | **t-1** | **t-2** | **t-1** | **t-2** | **t-1** | **t-2** | | **t-1** |
| **Sample Size** | **763** | | **3,851** | | **3,075** | | | **4,728** | |
| **Demographic** | | | | | | | | | |
| % Women | 48.3 (368) | | 51.8 (2,079) | | 49.6 (1,834) | | | 52.7 (2,502) | |
| Mean Age (SD) | - | - | - | - | - | - | 66.5 (9.22) | | 68.6 (9.15) |
| % Black, Asian, and Minority Ethnic | - | - | - | - | - | - | 4.8 (115) | | |
| **Education** | | | | | | | | | |
| % Degree | 10.8 (82) | 10.8 (82) | 4.8 (224) | 4.8 (244) | 7.1 (327) | 7.2 (335) | 18.1 (923) | | 21.7 (1,116) |
| **Socio-economic** | | | | | | | | | |
| % Self-reported financial difficulties | 5.3 (40) | 2.8 (21) | 6.7 (193) | 6.7 (197) | 8.7 (205) | 6.0 (123) | 23.0 (774) | | 18.8 (640) |
| % Homeowner (with/out mortgage) | 91.1 (695) | 96.5 (736) | 85.7 (3,447) | 86.7 (3,507) | 74.6 (2,546) | 74.6 (2,546) | 84.5 (3,956) | | 84.5 (3,964) |
| Occupational status/social class  % Managerial  % Intermediate  % Manual  % Other | 10.0 (76)  69.3 (528)  11.7 (90)  9.0 (69) | 10.0 (76)  69.3 (528)  11.7 (90)  9.0 (69) | 42.6 (1,879)  33.5 (1,215)  10.8 (351)  13.2 (401) | 47.3 (2,094)  29.8 (1,075)  15.1 (478)  7.9 (191) | 45.2 (1,581)  18.6 (595)  20.7 (564)  15.4 (335) | 43.9 (1,567)  20.4 (620)  20.4 (535)  15.3 (353) | 29.3 (1,622)  22.4 (1,145)  34.1 (1,394)  14.2 (279) | | 29.8 (1,623)  22.8 (1,158)  34.7 (1,411)  12.7 (248) |
| **Physical health** | | | | | | | | | |
| Self-reported health - % Poor/Fair | 6.8 (52) | 12.1 (92) | 14.5 (440) | 15.0 (432) | 16.0 (323) | 21.4 (434) | 24.3 (938) | | 25.9 (999) |
| % Limiting long-standing illness/health problem | 17.1 (130) | 20.2 (154) | 12.1 (362) | 14.2 (429) | 18.2 (413) | 21.1 (498) | 31.8 (1,358) | | 33.7 (1,465) |
| **Mental health** | | | | | | | | | |
| Life Satisfaction – Mean (SD) | 5.6 (1.33) | 5.6 (1.33) | 7.38 (1.66) | 7.37 (1.62) | 1.21 ( | 7.38 (1.67) | 7.28 (2.14) | | 7.44 (2.24) |
| Psychological distress – Mean (SD) | 1.21 (0.60) | 1.14 (0.42) | 1.45 (1.62) | 1.36 (1.74) | 1.85 (1.85) | 1.85 (1.85) | 1.31 (1.82) | | 1.35 (1.81) |

Note. NSHD = 1946 MRC National Survey of Health and Development; NCDS = 1958 National Child Development Study; BCS = 1970 British Cohort Study; ELSA = English Longitudinal Study of Ageing; UCLA = UCLA Loneliness Scale. Weighted %. Except for sex and ethnicity, all measures in each column refer to t-2 (time point before the most recent sweep before the COVID-19 survey) and t-1 (the most recent time point before the COVID-19 survey), where t represents the COVID-19 time point.

# **Supplementary file S2. Variables harmonization**

| ***Variables*** | ***Study*** | ***Variable code*** | ***Recoding if needed*** | ***Notes*** |
| --- | --- | --- | --- | --- |
| Social Isolation |  |  |  |  |
| *Household Size* - total in the household including CM |  |  |  |  |
|  | NSHD NCDS BCS | CW1_HHNUM | e.g., NCDS 9/11=8 | Recode to cap highest number in the household to ensure that anonymity is protects i.e., those with Ns under 10 |
|  | ELSA | CvNumP (at Covid Wave1) - How many people (including you) are currently living in the residence you are staying? |  | Values in sample range from 0 to 9 |
| *Living Alone* 0=Not Living Alone;1=Living Alone |  |  |  |  |
|  | NSHD NCDS BCS | CW1_HHNUM | e.g., NCDS 1/8=1 |  |
|  | ELSA | CvNumP | 2/max=0 |  |
| *Family Network* - Children, in household, outside of household, any. 0=No Children; 1=Any Children |  |  |  |  |
|  | NSHD NCDS BCS | CW1_HHNUMWH_2 CW1_ANYCHNL | 1=1; 2=0, gen (HH_CHILD_CV19) 1=1; 2=0, gen (FR_CHILD_CV19) gen ANY_CHILD_CV19 = . replace ANY_CHILD_CV19 = 0 if HH_CHILD_CV19==0 & FR_CHILD_CV19==0 replace ANY_CHILD_CV19 = 1 if HH_CHILD_CV19==1 \| FR_CHILD_CV19==1 | Generate whatever is available if only within household, fine, but use the same naming convention so it is clear |
|  | ELSA | Not asked in Covid Waves - we use pre-pandemic data |  |  |
| *Family Network* - Partner, in the household, outside of household, any. 0=No Partner; 1=Any Partner |  |  |  |  |
|  | NSHD NCDS BCS | CW1_HHNUMWH_1 CW1_OTHRELA | 1=1; 2=0, gen (HH_CHILD_CV19) 1=1; 2=0, gen (FR_CHILD_CV19) gen ANY_CHILD_CV19 = . replace ANY_CHILD_CV19 = 0 if HH_CHILD_CV19==0 & FR_CHILD_CV19==0 replace ANY_CHILD_CV19 = 1 if HH_CHILD_CV19==1 \| FR_CHILD_CV19==1 | Generate whatever is available if only within household, fine, but use the same naming convention so it is clear |
|  | ELSA | PartInHH (derived) - Relationship with other members in family is 'Husband/ Wife/ Partner'. 1=Yes, -1=Not applicable (only 1 person in HH) | 0 if PartInHH=-1 |  |
| *Family and Friends* - weekly in person contact with friends/relatives. 0=No Weekly Contact; 1=Weekly Contact |  |  |  |  |
|  | NSHD NCDS BCS | CW1_SCON1 CW1_SCON2 CW1_SCON3 | 1/4=1; 5=0, gen (FR_FF_FRQ_CV19) recode CW1_SCON2 CW1_SCON3 (-8=.) (1/4=1) (5=0), gen (FR_REM_FRQ_CV19_1 FR_REM_FRQ_CV19_2) gen FR_REM_FRQ_CV19 = . replace FR_REM_FRQ_CV19 = 0 if FR_REM_FRQ_CV19_1==0 & FR_REM_FRQ_CV19_2==0 replace FR_REM_FRQ_CV19 = 1 if FR_REM_FRQ_CV19_1==1 \| FR_REM_FRQ_CV19_2==1 gen ANY_FR_FRQ_CV19 = . replace ANY_FR_FRQ_CV19 = 0 if FR_FF_FRQ_CV19==0 & FR_REM_FRQ_CV19==0 replace ANY_FR_FRQ_CV19 = 1 if FR_FF_FRQ_CV19==1 \| FR_REM_FRQ_CV19==1 | Generate whatever is available if only within household, fine, but use the same naming convention so it is clear |
|  | ELSA | N/A |  |  |
| *Family and Friends* - weekly remote contact with friends/relatives. 0=No Weekly Contact; 1=Weekly Contact |  |  |  |  |
|  | NSHD NCDS BCS | CW1_SCON1 CW1_SCON2 CW1_SCON3 | 1/4=1; 5=0, gen (FR_FF_FRQ_CV19) recode CW1_SCON2 CW1_SCON3 (-8=.) (1/4=1) (5=0), gen (FR_REM_FRQ_CV19_1 FR_REM_FRQ_CV19_2) gen FR_REM_FRQ_CV19 = . replace FR_REM_FRQ_CV19 = 0 if FR_REM_FRQ_CV19_1==0 & FR_REM_FRQ_CV19_2==0 replace FR_REM_FRQ_CV19 = 1 if FR_REM_FRQ_CV19_1==1 \| FR_REM_FRQ_CV19_2==1 gen ANY_FR_FRQ_CV19 = . replace ANY_FR_FRQ_CV19 = 0 if FR_FF_FRQ_CV19==0 & FR_REM_FRQ_CV19==0 replace ANY_FR_FRQ_CV19 = 1 if FR_FF_FRQ_CV19==1 \| FR_REM_FRQ_CV19==1 | Generate whatever is available if only within household, fine, but use the same naming convention so it is clear |
|  | ELSA | FAM: In the past month, how often have you done the following with any of your immediate family (parents, children, grandchildren and brothers and sisters), not counting any who live with you? FRD: In the past month, how often have you done the following with other relatives and/or friends?   1. Speak on the phone -- 1.Daily; 2.Three to Six times a week; 3.Once/Twice a week; 4.Less than once a week or never  2. Write/ email -- 1.Daily; 2.Three to Six times a week; 3.Once/Twice a week; 4.Less than once a week or never  3. Send/ receive texts -- 1.Daily; 2.Three to Six times a week; 3.Once/Twice a week; 4.Less than once a week or never | 0 (no weekly contact) if (fam1==4 & fam2==4 & fam3==4) & (frd1==4& frd2==4 & frd3==4) |  |
| *Education and employment* - neither in education nor employment. 0=Neither in Education Nor Employment; 1=In Education or Employment |  |  |  |  |
|  | NSHD NCDS BCS | CW1_ECONACTIVITYD | 1=1; 2/4=0; 5=1; 6/9=0; 10=1; 11/13=0 |  |
|  | ELSA | CvPstd. Which of the following would you say best describes your current situation?  1.Retired; 2.Employed; 3.Paid/unpaid leave from employment (including furlough); 4.Self-employed and currently working; 5.Self-employed but not currently working; 6.Unemployed; 7.Permanently sick or disabled; 8.Looking after home or family | 1 if CvPstd==2 \| CvPstd_w1==4 0 for all other categories |  |
| *Community engagement* - membership to club/organisation or volunteering. 0=No Community Engagement; 1=Community Engagement |  |  |  |  |
|  | NSHD NCDS BCS |  | 1/4=1; 5=0 |  |
|  | ELSA | CvVolun. Have you changed the frequency you take part in voluntary work due to the coronavirus outbreak? 1. Yes, stopped completely; 2.Yes, less than before; 3.Yes, more than before; 4.No, about the same; 5.I did not volunteer previously | 1 if CvVolun==2 OR 3 OR 4 0 if CvVolun==1 OR 5 |  |
| *Overall social isolation indicator* 0-6; 0=Not Socially Isolated; 1=Socially Isolated |  |  |  |  |
|  | NSHD NCDS BCS |  | gen SI_CV19 = . replace SI_CV19 = 0 if HH_ALONE_CV19==1 & FR_FF_FRQ_CV19==0 & EE_SIZ_CV19_DUR==0 & /// COM_FRQ_CV19==0 & COM_VOL_CV19==0 replace SI_CV19 = 1 if HH_ALONE_CV19==0 \| FR_FF_FRQ_CV19==1 \| EE_SIZ_CV19_DUR==1 \| /// COM_FRQ_CV19==1 \| COM_VOL_CV19==1 |  |
| Loneliness |  |  |  |  |
| *UCLA Loneliness Scale* - total score and binary 0=Not Lonely; 1=Lonely |  |  |  |  |
|  | NSHD NCDS BCS |  | recode CW1_LONELY_1 CW1_LONELY_2 CW1_LONELY_3 CW1_LONELY_4 (-8=.), gen (LONE_CV19_1 LONE_CV19_2 LONE_CV19_3 LONE_CV19_4) egen LONE_TOTAL_CV19 = rowtotal(LONE_CV19_1 LONE_CV19_2 LONE_CV19_3) , missing gen LONE_CV19_01 = . replace LONE_CV19_01 = 0 if LONE_TOTAL_CV19==3 \| LONE_TOTAL_CV19==4 \| LONE_TOTAL_CV19==5 replace LONE_CV19_01 = 1 if LONE_TOTAL_CV19==6 \| LONE_TOTAL_CV19==7 \| LONE_TOTAL_CV19==8 \| LONE_TOTAL_CV19==9 gen LONE_CV19_4_01 = . replace LONE_CV19_4_01 = 0 if LONE_CV19_4==1 replace LONE_CV19_4_01 = 1 if LONE_CV19_4==2 \| LONE_CV19_4==3 |  |
|  | ELSA | A. How often do you feel you lack companionship? B. How often do you feel left out? C. How often do you feel isolated from others? D. How often do you feel lonely?  -- 1.Hardly Ever or Never; 2.Some of the time; 3.Often | For each question, 'lonely' if ==3  For UCLA total score: (mean of all scores)*3 If value is equal or greater than 6 --> Lonely |  |

# **Supplementary file S3. Networks by age-bands in ELSA**

#

# **Supplementary file S4. Tetrachoric correlation matrices**

## NCDS pre-COVID

|  | LoneHH | Child | Part | Frnd | NEET | Comm | Lonely |
| --- | --- | --- | --- | --- | --- | --- | --- |
| LoneHH | 1 | 0.589983 | 0.981843 | -0.02602 | 0.042372 | 0.076241 | 0.135251 |
| Child | 0.589983 | 1 | 0.457515 | 0.073806 | -0.03365 | 0.025242 | 0.056223 |
| Part | 0.981843 | 0.457515 | 1 | -0.00374 | 0.098771 | 0.037865 | 0.091211 |
| Frnd | -0.02602 | 0.073806 | -0.00374 | 1 | -0.14037 | 0.12262 | 0.115433 |
| NEET | 0.042372 | -0.03365 | 0.098771 | -0.14037 | 1 | 0.039855 | 0.411054 |
| Comm | 0.076241 | 0.025242 | 0.037865 | 0.12262 | 0.039855 | 1 | 0.060861 |
| Lonely | 0.135251 | 0.056223 | 0.091211 | 0.115433 | 0.411054 | 0.060861 | 1 |

## NCDS COVID

|  | LoneHH | Child | Part | Frnd | NEET | Comm | Lonely |
| --- | --- | --- | --- | --- | --- | --- | --- |
| LoneHH | 1 | 0.437798 | 0.751395 | -0.03528 | -0.08684 | 0.060672 | 0.317671 |
| Child | 0.437798 | 1 | 0.378367 | 0.01977 | 0.097583 | 0.052744 | 0.062364 |
| Part | 0.751395 | 0.378367 | 1 | -0.07829 | -0.01609 | 0.082119 | 0.328169 |
| Frnd | -0.03528 | 0.01977 | -0.07829 | 1 | -0.05501 | 0.21568 | 0.016342 |
| NEET | -0.08684 | 0.097583 | -0.01609 | -0.05501 | 1 | 0.006817 | 0.196053 |
| Comm | 0.060672 | 0.052744 | 0.082119 | 0.21568 | 0.006817 | 1 | 0.124797 |
| Lonely | 0.317671 | 0.062364 | 0.328169 | 0.016342 | 0.196053 | 0.124797 | 1 |

## BCS pre-COVID

|  | LoneHH | Child | Part | Frnd | NEET | Comm | Lonely |
| --- | --- | --- | --- | --- | --- | --- | --- |
| LoneHH | 1 | 0.397456 | 0.602432 | -0.05752 | 0.408745 | 0.106978 | 0.40521 |
| Child | 0.397456 | 1 | 0.185457 | 0.098693 | 0.068008 | 0.04436 | 0.016794 |
| Part | 0.602432 | 0.185457 | 1 | -0.13935 | 0.351058 | 0.080645 | 0.283281 |
| Frnd | -0.05752 | 0.098693 | -0.13935 | 1 | -0.01185 | 0.158659 | 0.007892 |
| NEET | 0.408745 | 0.068008 | 0.351058 | -0.01185 | 1 | 0.227435 | 0.3624 |
| Comm | 0.106978 | 0.04436 | 0.080645 | 0.158659 | 0.227435 | 1 | 0.286927 |
| Lonely | 0.40521 | 0.016794 | 0.283281 | 0.007892 | 0.3624 | 0.286927 | 1 |

## BCS COVID

|  | LoneHH | Child | Part | Frnd | NEET | Comm | Lonely |
| --- | --- | --- | --- | --- | --- | --- | --- |
| LoneHH | 1 | 0.540456 | 0.607044 | -0.12761 | 0.144962 | 0.064402 | 0.449424 |
| Child | 0.540456 | 1 | 0.326816 | 0.053917 | 0.020345 | 0.093774 | 0.13131 |
| Part | 0.607044 | 0.326816 | 1 | -0.01306 | 0.183432 | 0.032101 | 0.405652 |
| Frnd | -0.12761 | 0.053917 | -0.01306 | 1 | 0.053566 | 0.161491 | 0.058226 |
| NEET | 0.144962 | 0.020345 | 0.183432 | 0.053566 | 1 | 0.117307 | 0.385055 |
| Comm | 0.064402 | 0.093774 | 0.032101 | 0.161491 | 0.117307 | 1 | 0.251157 |
| Lonely | 0.449424 | 0.13131 | 0.405652 | 0.058226 | 0.385055 | 0.251157 | 1 |

## ELSA Age 50 Pre-COVID

|  | LoneHH | Child | Part | Frnd | NEET | Comm | Comp | Left | Iso | Lonely |
| --- | --- | --- | --- | --- | --- | --- | --- | --- | --- | --- |
| LoneHH | 1 | 0.451849 | 0.939875 | -0.45286 | 0.353296 | -0.10794 | 0.276963 | 0.354281 | 0.314474 | 0.320358 |
| Child | 0.451849 | 1 | 0.414935 | -0.45795 | 0.190906 | -0.08095 | 0.244248 | 0.036647 | 0.259694 | 0.187217 |
| Part | 0.939875 | 0.414935 | 1 | -0.51836 | 0.287206 | 0.045539 | 0.447002 | 0.350625 | 0.304802 | 0.40931 |
| Frnd | -0.45286 | -0.45795 | -0.51836 | 1 | -0.50598 | 0.31161 | -0.24449 | -0.24965 | 0.00585 | -0.06043 |
| NEET | 0.353296 | 0.190906 | 0.287206 | -0.50598 | 1 | -0.06887 | 0.400835 | 0.582055 | 0.489074 | 0.360708 |
| Comm | -0.10794 | -0.08095 | 0.045539 | 0.31161 | -0.06887 | 1 | 0.374102 | -0.09162 | 0.16569 | 0.032271 |
| Comp | 0.276963 | 0.244248 | 0.447002 | -0.24449 | 0.400835 | 0.374102 | 1 | 0.774116 | 0.83787 | 0.863883 |
| Left | 0.354281 | 0.036647 | 0.350625 | -0.24965 | 0.582055 | -0.09162 | 0.774116 | 1 | 0.874752 | 0.823307 |
| Iso | 0.314474 | 0.259694 | 0.304802 | 0.00585 | 0.489074 | 0.16569 | 0.83787 | 0.874752 | 1 | 0.890673 |
| Lonely | 0.320358 | 0.187217 | 0.40931 | -0.06043 | 0.360708 | 0.032271 | 0.863883 | 0.823307 | 0.890673 | 1 |

ELSA Age 60 Pre-COVID

|  | LoneHH | Child | Part | Frnd | NEET | Comm | Comp | Left | Iso | Lonely |
| --- | --- | --- | --- | --- | --- | --- | --- | --- | --- | --- |
| LoneHH | 1 | 0.383085 | 0.978913 | -0.09568 | 0.01911 | -0.03119 | 0.549765 | 0.235998 | 0.323824 | 0.452652 |
| Child | 0.383085 | 1 | 0.287047 | -0.47519 | 0.062498 | -0.11484 | -0.00582 | -0.08846 | -0.02812 | -0.08428 |
| Part | 0.978913 | 0.287047 | 1 | -0.09239 | 0.04595 | 0.024217 | 0.568407 | 0.308607 | 0.38639 | 0.535454 |
| Frnd | -0.09568 | -0.47519 | -0.09239 | 1 | -0.06384 | -0.01628 | 0.031976 | 0.091371 | 0.2473 | 0.011792 |
| NEET | 0.01911 | 0.062498 | 0.04595 | -0.06384 | 1 | -0.18456 | 0.222777 | 0.279074 | 0.351702 | 0.276801 |
| Comm | -0.03119 | -0.11484 | 0.024217 | -0.01628 | -0.18456 | 1 | 0.163964 | 0.135114 | 0.052945 | 0.110457 |
| Comp | 0.549765 | -0.00582 | 0.568407 | 0.031976 | 0.222777 | 0.163964 | 1 | 0.822769 | 0.781851 | 0.92259 |
| Left | 0.235998 | -0.08846 | 0.308607 | 0.091371 | 0.279074 | 0.135114 | 0.822769 | 1 | 0.854579 | 0.867267 |
| Iso | 0.323824 | -0.02812 | 0.38639 | 0.2473 | 0.351702 | 0.052945 | 0.781851 | 0.854579 | 1 | 0.883253 |
| Lonely | 0.452652 | -0.08428 | 0.535454 | 0.011792 | 0.276801 | 0.110457 | 0.92259 | 0.867267 | 0.883253 | 1 |

## ELSA Age 70 pre-COVID

|  | LoneHH | Child | Part | Frnd | NEET | Comm | Comp | Left | Iso | Lonely |
| --- | --- | --- | --- | --- | --- | --- | --- | --- | --- | --- |
| LoneHH | 1 | 0.399645 | 0.974446 | -0.16624 | 0.04001 | 0.052877 | 0.47768 | 0.265956 | 0.128811 | 0.430595 |
| Child | 0.399645 | 1 | 0.369355 | -0.42353 | 0.02554 | -0.01641 | 0.209588 | 0.099042 | 0.045297 | 0.216326 |
| Part | 0.974446 | 0.369355 | 1 | -0.09259 | 0.012459 | 0.088588 | 0.485734 | 0.262194 | 0.1555 | 0.429922 |
| Frnd | -0.16624 | -0.42353 | -0.09259 | 1 | 0.198287 | -0.12825 | 0.136949 | 0.165375 | -0.02239 | 0.111558 |
| NEET | 0.04001 | 0.02554 | 0.012459 | 0.198287 | 1 | -0.10868 | -0.00156 | 0.095476 | 0.080671 | 0.061863 |
| Comm | 0.052877 | -0.01641 | 0.088588 | -0.12825 | -0.10868 | 1 | 0.053688 | 0.228344 | 0.068298 | 0.138214 |
| Comp | 0.47768 | 0.209588 | 0.485734 | 0.136949 | -0.00156 | 0.053688 | 1 | 0.772677 | 0.806247 | 0.879767 |
| Left | 0.265956 | 0.099042 | 0.262194 | 0.165375 | 0.095476 | 0.228344 | 0.772677 | 1 | 0.863761 | 0.766242 |
| Iso | 0.128811 | 0.045297 | 0.1555 | -0.02239 | 0.080671 | 0.068298 | 0.806247 | 0.863761 | 1 | 0.79257 |
| Lonely | 0.430595 | 0.216326 | 0.429922 | 0.111558 | 0.061863 | 0.138214 | 0.879767 | 0.766242 | 0.79257 | 1 |

ELSA Age 80 pre-COVID

|  | LoneHH | Child | Part | Frnd | NEET | Comm | Comp | Left | Iso | Lonely |
| --- | --- | --- | --- | --- | --- | --- | --- | --- | --- | --- |
| LoneHH | 1 | 0.320526 | 0.966707 | -0.3238 | 0.01714 | -0.07537 | 0.420509 | 0.164338 | 0.177097 | 0.558602 |
| Child | 0.320526 | 1 | 0.328673 | -0.2977 | -0.04835 | -0.01623 | -0.09973 | 0.121302 | 0.078793 | -0.15653 |
| Part | 0.966707 | 0.328673 | 1 | -0.17532 | 0.080824 | -0.00057 | 0.418046 | 0.223032 | 0.195403 | 0.534549 |
| Frnd | -0.3238 | -0.2977 | -0.17532 | 1 | -0.10871 | 0.12524 | 0.189284 | 0.353869 | 0.060397 | -0.06802 |
| NEET | 0.01714 | -0.04835 | 0.080824 | -0.10871 | 1 | 0.045108 | -0.069 | -0.11976 | -0.13262 | -0.08319 |
| Comm | -0.07537 | -0.01623 | -0.00057 | 0.12524 | 0.045108 | 1 | 0.300684 | 0.164827 | 0.065193 | 0.166479 |
| Comp | 0.420509 | -0.09973 | 0.418046 | 0.189284 | -0.069 | 0.300684 | 1 | 0.671157 | 0.630271 | 0.873776 |
| Left | 0.164338 | 0.121302 | 0.223032 | 0.353869 | -0.11976 | 0.164827 | 0.671157 | 1 | 0.692109 | 0.692551 |
| Iso | 0.177097 | 0.078793 | 0.195403 | 0.060397 | -0.13262 | 0.065193 | 0.630271 | 0.692109 | 1 | 0.619083 |
| Lonely | 0.558602 | -0.15653 | 0.534549 | -0.06802 | -0.08319 | 0.166479 | 0.873776 | 0.692551 | 0.619083 | 1 |

## ELSA Age 50 COVID

|  | LoneHH | Child | Part | Frnd | NEET | Comm | Comp | Left | Iso | Lonely |
| --- | --- | --- | --- | --- | --- | --- | --- | --- | --- | --- |
| LoneHH | 1 | 0.526352 | 0.945342 | -0.30334 | 0.453324 | 0.075848 | 0.535604 | 0.430429 | 0.690622 | 0.546878 |
| Child | 0.526352 | 1 | 0.434588 | -0.06617 | 0.11973 | -0.04773 | 0.206936 | 0.346076 | 0.282863 | 0.215577 |
| Part | 0.945342 | 0.434588 | 1 | -0.29054 | 0.335664 | 0.014807 | 0.483178 | 0.321416 | 0.579576 | 0.516607 |
| Frnd | -0.30334 | -0.06617 | -0.29054 | 1 | -0.30605 | 0.137356 | 0.551555 | 0.562314 | -0.09705 | 0.477383 |
| NEET | 0.453324 | 0.11973 | 0.335664 | -0.30605 | 1 | 0.150407 | 0.240969 | 0.202022 | 0.53946 | 0.392409 |
| Comm | 0.075848 | -0.04773 | 0.014807 | 0.137356 | 0.150407 | 1 | 0.138004 | 0.064083 | 0.318021 | 0.374614 |
| Comp | 0.535604 | 0.206936 | 0.483178 | 0.551555 | 0.240969 | 0.138004 | 1 | 0.890407 | 0.641802 | 0.907804 |
| Left | 0.430429 | 0.346076 | 0.321416 | 0.562314 | 0.202022 | 0.064083 | 0.890407 | 1 | 0.690864 | 0.85969 |
| Iso | 0.690622 | 0.282863 | 0.579576 | -0.09705 | 0.53946 | 0.318021 | 0.641802 | 0.690864 | 1 | 0.760406 |
| Lonely | 0.546878 | 0.215577 | 0.516607 | 0.477383 | 0.392409 | 0.374614 | 0.907804 | 0.85969 | 0.760406 | 1 |

ELSA Age 60 COVID

|  | LoneHH | Child | Part | Frnd | NEET | Comm | Comp | Left | Iso | Lonely |
| --- | --- | --- | --- | --- | --- | --- | --- | --- | --- | --- |
| LoneHH | 1 | 0.330398 | 0.973276 | 0.144898 | -0.00581 | 0.109869 | 0.487175 | 0.319536 | 0.289733 | 0.407375 |
| Child | 0.330398 | 1 | 0.266736 | 0.29848 | 0.03422 | -0.07286 | -0.01317 | 0.035607 | -0.06055 | -0.08771 |
| Part | 0.973276 | 0.266736 | 1 | 0.031978 | -0.01171 | 0.141829 | 0.507257 | 0.313698 | 0.355906 | 0.382725 |
| Frnd | 0.144898 | 0.29848 | 0.031978 | 1 | 0.127674 | 0.179484 | 0.183727 | 0.150305 | 0.016903 | 0.197825 |
| NEET | -0.00581 | 0.03422 | -0.01171 | 0.127674 | 1 | 0.074739 | 0.149956 | 0.138083 | 0.235699 | 0.223644 |
| Comm | 0.109869 | -0.07286 | 0.141829 | 0.179484 | 0.074739 | 1 | 0.302476 | -0.03924 | 0.162769 | 0.024973 |
| Comp | 0.487175 | -0.01317 | 0.507257 | 0.183727 | 0.149956 | 0.302476 | 1 | 0.834481 | 0.761324 | 0.842114 |
| Left | 0.319536 | 0.035607 | 0.313698 | 0.150305 | 0.138083 | -0.03924 | 0.834481 | 1 | 0.853745 | 0.838118 |
| Iso | 0.289733 | -0.06055 | 0.355906 | 0.016903 | 0.235699 | 0.162769 | 0.761324 | 0.853745 | 1 | 0.834776 |
| Lonely | 0.407375 | -0.08771 | 0.382725 | 0.197825 | 0.223644 | 0.024973 | 0.842114 | 0.838118 | 0.834776 | 1 |

ELSA Age 70 COVID

|  | LoneHH | Child | Part | Frnd | NEET | Comm | Comp | Left | Iso | Lonely |
| --- | --- | --- | --- | --- | --- | --- | --- | --- | --- | --- |
| LoneHH | 1 | 0.345508 | 0.994649 | -0.12214 | -0.00807 | 0.129009 | 0.450973 | 0.29103 | 0.199965 | 0.369476 |
| Child | 0.345508 | 1 | 0.321967 | 0.220415 | 0.102659 | 0.010607 | 0.19631 | 0.169037 | 0.008588 | 0.118528 |
| Part | 0.994649 | 0.321967 | 1 | -0.05076 | -0.0209 | 0.134498 | 0.443447 | 0.318151 | 0.217263 | 0.380893 |
| Frnd | -0.12214 | 0.220415 | -0.05076 | 1 | 0.078377 | 0.043453 | -0.04835 | 0.149143 | 0.0684 | 0.019326 |
| NEET | -0.00807 | 0.102659 | -0.0209 | 0.078377 | 1 | 0.056355 | 0.004408 | 0.162927 | 0.047568 | 0.096965 |
| Comm | 0.129009 | 0.010607 | 0.134498 | 0.043453 | 0.056355 | 1 | 0.067263 | 0.108393 | 0.053713 | 0.127339 |
| Comp | 0.450973 | 0.19631 | 0.443447 | -0.04835 | 0.004408 | 0.067263 | 1 | 0.857882 | 0.742761 | 0.886924 |
| Left | 0.29103 | 0.169037 | 0.318151 | 0.149143 | 0.162927 | 0.108393 | 0.857882 | 1 | 0.880747 | 0.854633 |
| Iso | 0.199965 | 0.008588 | 0.217263 | 0.0684 | 0.047568 | 0.053713 | 0.742761 | 0.880747 | 1 | 0.770768 |
| Lonely | 0.369476 | 0.118528 | 0.380893 | 0.019326 | 0.096965 | 0.127339 | 0.886924 | 0.854633 | 0.770768 | 1 |

## ELSA Age 80 COVID

|  | LoneHH | Child | Part | Frnd | NEET | Comm | Comp | Left | Iso | Lonely |
| --- | --- | --- | --- | --- | --- | --- | --- | --- | --- | --- |
| LoneHH | 1 | 0.292347 | 0.978011 | -0.2649 | -0.31828 | -0.02852 | 0.576592 | 0.424378 | 0.3541 | 0.522757 |
| Child | 0.292347 | 1 | 0.351486 | 0.269162 | -0.22576 | -0.09649 | -0.10341 | -0.23734 | -0.44715 | 0.054253 |
| Part | 0.978011 | 0.351486 | 1 | -0.15908 | -0.24799 | -0.06333 | 0.493014 | 0.337039 | 0.248731 | 0.48098 |
| Frnd | -0.2649 | 0.269162 | -0.15908 | 1 | -0.05845 | 0.25755 | -0.35474 | -0.0579 | -0.20985 | -0.06607 |
| NEET | -0.31828 | -0.22576 | -0.24799 | -0.05845 | 1 | 0.155005 | 0.026979 | -0.10043 | 0.040525 | -0.07779 |
| Comm | -0.02852 | -0.09649 | -0.06333 | 0.25755 | 0.155005 | 1 | -0.05811 | -0.03848 | 0.070941 | -0.09663 |
| Comp | 0.576592 | -0.10341 | 0.493014 | -0.35474 | 0.026979 | -0.05811 | 1 | 0.689218 | 0.663719 | 0.857553 |
| Left | 0.424378 | -0.23734 | 0.337039 | -0.0579 | -0.10043 | -0.03848 | 0.689218 | 1 | 0.889344 | 0.781213 |
| Iso | 0.3541 | -0.44715 | 0.248731 | -0.20985 | 0.040525 | 0.070941 | 0.663719 | 0.889344 | 1 | 0.709647 |
| Lonely | 0.522757 | 0.054253 | 0.48098 | -0.06607 | -0.07779 | -0.09663 | 0.857553 | 0.781213 | 0.709647 | 1 |
